# International Studies of Prenatal Exposure to Polycyclic Aromatic Hydrocarbons and Fetal Growth

**DOI:** 10.1289/ehp.8982

**Published:** 2006-08-03

**Authors:** Hyunok Choi, Wieslaw Jedrychowski, John Spengler, David E. Camann, Robin M. Whyatt, Virginia Rauh, Wei-Yann Tsai, Frederica P. Perera

**Affiliations:** 1 Columbia Center for Children's Environmental Health, Mailman School of Public Health, Columbia University, New York, New York, USA; 2 Epidemiology and Preventive Medicine, College of Medicine, Jagiellonian University, Krakow, Poland; 3 Department of Environmental Health, Harvard School of Public Health, Boston, Massachusetts, USA; 4 Department of Analytical and Environmental Chemistry, Southwest Research Institute, San Antonio, Texas USA; 5 Department of Biostatistics, Mailman School of Public Health, Columbia University, New York, New York, USA; 6 Department of Statistics, National Cheng Kung University, Taiwan

**Keywords:** birth outcomes, birth weight, fetal toxicity, personal air monitoring, polycyclic aromatic hydrocarbons, prenatal

## Abstract

**Objectives:**

Polycyclic aromatic hydrocarbons (PAHs) are ubiquitously distributed human mutagens and carcinogens. However, lack of adequate air monitoring data has limited understanding of the effects of airborne PAHs on fetal growth. To address this gap in knowledge, we examined the association between prenatal exposure to airborne PAHs and birth weight, birth length, and birth head circumference, respectively, in Krakow, Poland, and New York City (NYC).

**Methods:**

The parallel prospective cohort studies enrolled nonsmoking, healthy, and nonoccupationally exposed women and their newborns. Personal air monitoring of pregnant women was conducted over 48 hr. To control for maternal environmental tobacco smoke (ETS) exposure, we excluded those with umbilical cord plasma cotinine concentrations > 25 ng/mL. Mean cord plasma cotinine concentrations in both ethnic groups were ≤ 0.5 ng/mL.

**Results:**

Prenatal PAH exposure was 10-fold higher in Krakow than in NYC. Prenatal PAH exposure was associated with significantly reduced birth weight in both Krakow Caucasians (*p* < 0.01) and in NYC African Americans (*p* < 0.01), controlling for known and potential confounders, but not in NYC Dominicans. Within the lower exposure range common to the two cities (1.80–36.47 ng/m^3^), the effect per unit PAH exposure on birth weight was 6-fold greater for NYC African Americans than for Krakow Caucasians (*p* = 0.01).

**Conclusions:**

These results confirm the adverse reproductive effect of relatively low PAH concentrations in two populations and suggest increased susceptibility of NYC African Americans. Fetal growth impairment has been linked to child developmental and health problems. Thus, substantial health benefits would result from global reduction of PAH emissions.

Polycyclic aromatic hydrocarbons (PAHs) are ubiquitous air pollutants generated by combustion sources that include diesel- and gasoline-powered motor vehicles, coal-fired power plants, residential heating, cooking, and tobacco smoking (Bostrom et al. 2002). An estimated > 80% of airborne PAHs results from the combustion of fossil fuels and biomass (Bostrom et al. 2002). There is growing concern about the adverse effects of air pollution from fossil fuel combustion on public health (Bostrom et al. 2002; Brunekreef and Holgate 2002) and the global climate (National Academy of Sciences 2001). PAHs are human mutagens and carcinogens (Bostrom et al. 2002) and are potentially significant reproductive and developmental toxicants (Dejmek et al. 2000; Perera et al. 2003). For example, prenatal exposure of rats to 25–100 μg/m^3^ of the representative PAH, benzo[*a*]pyrene (BaP), through maternal inhalation significantly decreased the fetal survival rate and birth weight in a dose-dependent manner (Archibong et al. 2002). In humans, associations between PAHs or PAH–DNA damage and fetal growth reduction have been reported in some but not all studies (Dejmek et al. 2000; Perera et al. 1998, 2003; Šrám et al. 2005). In the Czech Republic, ambient PAHs significantly increased the risk of intrauterine growth retardation (IUGR) both in Teplice and Prachatice (Dejmek et al. 2000). An earlier study in Poland found that the infants who had higher than median leukocyte PAH–DNA adduct level had significantly reduced birth weight, length, and head circumference (Perera et al. 1998). In New Jersey, USA, the risk of fetal death, premature birth, and low birth weight was significantly higher for those with a high prenatal polycyclic organic matter exposure (Vassilev et al. 2001). However, the limitations of most prior studies [retrospective or cross-sectional design, reliance on ambient air monitoring data, failure to account for co-exposure to other sources of PAHs such as environmental tobacco smoke (ETS)] have prevented definitive causal inferences regarding airborne PAHs and impaired fetal growth (Šrám et al. 2005).

We conducted two parallel prospective cohort studies in Krakow, Poland, and New York City (NYC), USA, using identical eligibility criteria, study design, and personal air monitoring methodology. Here we examined effects across the entire range of PAH exposure in each cohort separately, to understand the relationship between *in utero* exposure to airborne PAHs and fetal growth within each population. We also conducted exploratory analyses comparing the magnitude of PAH-related effects between the two different populations within the exposure range common to both, recognizing the limitations of such international, interethnic comparisons.

The U.S. and Polish cohorts represent a wide range of exposure, experienced in many other urban areas; therefore, study results in Krakow and NYC are relevant to other populations worldwide. In the Krakow cohort, the average PAH concentration estimated by personal air monitoring was 39.0 ng/m^3^ (range, 1.8–272.2 ng/m^3^), compared with 3.3 ng/m^3^ (range, 0.3–36.5 ng/m^3^) in NYC. Comparable personal air monitoring data on PAHs are not available for other populations, but stationary ambient air monitoring data for BaP can be used for comparison. The mean ambient BaP level in Krakow was 3.8 ng/m^3^ in 2000. Annual mean ambient BaP level in various locations within Krakow ranged from 4 to 10 ng/m^3^ during 2002. This level is comparable to the winter means reported in Teplice (7.42 ng/m^3^) and Prachatice (5.37 ng/m^3^), Czech Republic, between 1993 and 1994 (Binková et al. 1999) but lower than that measured in Tongliang, China, between 2002 and 2003 (15 ng/m^3^) (Chow et al. 2006). In an ongoing prospective study in NYC, personal exposure to BaP among the cohort of pregnant women was 0.49 ± 0.65 ng/m^3^ (Tonne et al. 2004). In U.S. cities, as well as in many urban areas of Europe, ambient BaP concentrations are generally < 1 ng/m^3^, but may range between 1 and 5 ng/m^3^ during winter or in areas with heavy traffic (Naumova et al. 2002; Tonne et al. 2004).

## Methods

### Subjects: Krakow study

Details on study design for the Krakow cohort have been previously published (Jedrychowski et al. 2004, 2006). Nonsmoking, pregnant women residing in the Srodmiescie (Old Podgorze) and the Krowodrza-Nowa Huta (New Podgorze) areas were recruited between November 2000 and March 2003 (Jedrychowski et al. 2004). Pregnant women were eligible if they were not currently smoking, registered at prenatal health care clinics in either of the two target areas, had lived at the present address for at least a year before the initial interview, were between the 8th and 24th weeks of gestation, were ≥ 18 years of age, had no current occupational exposure to PAHs or other known developmental toxicants, had no history of illicit drug use, pregnancy-related diabetes, or hypertension, and had a valid estimate of gestational age. Between the 20th and 30th week of pregnancy, research workers administered in-depth health, lifestyle, and environmental exposure (HLEE) questionnaires to women in their homes (Jedrychowski et al. 2004; Perera et al. 2003). On completing the interview, 48-hr personal air monitoring was carried out as described (Jedrychowski et al. 2004; Perera et al. 2003). To examine consistency in self-reporting, the interview was repeated during the third trimester. After delivery, data on pregnancy and delivery were obtained from the mothers’ and infants’ medical records. Informed consent was obtained from all subjects and the study was approved by the ethics committee of the Jagiellonian University.

### Subjects: NYC study

Details on study design and results on the initial 263 subjects for the NYC cohort have been previously published (Perera et al. 2003). The population residing in Washington Heights, Harlem, and the South Bronx is predominantly African American and Dominican. As previously described, women were recruited at the time of the first prenatal visit and were monitored during the third trimester (Perera et al. 2003). Ethnicity was self-identified. The same protocol as that for the Krakow cohort was used to conduct HLEE interviews, monitor personal exposure to PAHs, and collect birth outcome data. The present report includes 380 subjects as of September 2004, who met the inclusion criteria and had complete information on potential fetal growth confounders. The institutional review board of Columbia Presbyterian Medical Center approved the study, and informed consent was obtained from all study participants.

### Air monitoring

Details of the personal air monitoring conducted in Krakow (Jedrychowski et al. 2004, 2006) and NYC (Perera et al. 2003; Tonne et al. 2004) have been published. Briefly, on completion of the interview, the women were given a backpack containing a portable personal exposure air monitor to be worn during the day and kept near the bed at night during a consecutive 48-hr period. Personal air monitoring data were given a quality assurance (QA) score (0–3) for flow rate, flow time, and completeness of documentation (Kinney et al. 2002). A final QA score of 0 (highest quality) or 1 (high quality) was required for inclusion. For both cohorts, the air extracts were analyzed at Southwest Research Institute for levels of pyrene and eight carcinogenic PAHs (∑8 c-PAHs): benzo[*a*]anthracene, chrysene, benzo[*b*]fluoranthene, benzo[*k*]fluoranthene, benzo[*a*]pyrene, indeno[*1,2,3-cd*]pyrene, dibenz[*a,h*]anthracene, and benzo[*g,h,i*]perylene (Camaan and Whyatt 2001; Perera et al. 2003).

To examine the representativeness of a single 48-hr monitoring, a subset of the Krakow cohort was monitored once each trimester (*n* = 72, 72, and 68, respectively). However, the entire NYC cohort was monitored once. Unlike for NYC subjects, personal exposure to pesticides was not monitored for the Krakow cohort, based on the low residential pesticide use (Jedrychowski W, personal communication). Details of the blood sample collection for biomarker analysis have been reported (Perera et al. 2003; Rauh et al. 2004).

### Statistical analysis

Our primary goal was to examine the consistency of the airborne PAH–birth outcome association in the Krakow and the NYC cohorts, respectively. Subsequently, the data from the two studies were merged to compare the association across the ethnic groups. In Krakow and NYC, subjects with complete data on personally monitored PAHs, birth outcomes, and potential confounding variables were included. To preclude selection bias, the demographic characteristics of those included versus not included were examined with analysis of variance (ANOVA) for the continuous variables, followed by Bonferroni correction and pair-wise chi-square test for categorical variables. For both cohorts, the ∑8 c-PAHs was used as the independent variable because the PAHs were intercorrelated (Spearman’s rho = 0.96–0.98). We conducted the multivariate linear regression analysis using hierarchical backward elimination strategy (Kleinbaum and Klein 2002), while examining multicollinearity, multiple testing, and influential observations as possible sources of spurious association.

We conduction regression diagnostics before and after model specification. Influential observations (one African American and one Caucasian) were dropped. The birth outcomes, gestational age, cord cotinine, and ∑8 c-PAHs were natural-log (ln) transformed because their distributions significantly deviated from normality (Kolmogorov-Smirnov test *p*-values < 0.001). The following categorical variables were created from the interview data: maternal education (< high school; high school graduate; > high school); high dietary intake of PAHs during current pregnancy (smoked, grilled, barbequed/blackened food items ≥ 2/week); parity (≥ one prior singleton or multiple births); any consumption of wine, beer, or hard liquor, respectively; daily alcoholic intake (≥ one glass of wine, can of beer, or 2-oz shot of hard alcohol per day); marital status (married or cohabit with same partner ≥ 7 years), and maternal ETS exposure at home or at work (≥ one smoker; number of smokers; and number of cigarettes smoked in the woman’s presence), and prepregnancy weight and height. The season of delivery was defined as fall (September–November), winter (December–February), and spring (March–May), using summer (June–August) as the reference. To further validate non-smoking status, the subjects were restricted to those who had < 25 ng/mL cotinine in the umbilical cord serum. If the cord cotinine was below the limit of detection (LOD) (0.025 ng/mL), the value of LOD/2 was attributed to that sample. Whenever the newborn’s value was missing, the maternal level was used because there is a high correlation between cord and maternal blood concentrations.

We validated the reliability of the gestational age obtained from the infant’s medical record by independently deriving the gestational age with date of last menstrual period (LMP) and/or sonogram. LMP-based gestational age was calculated as


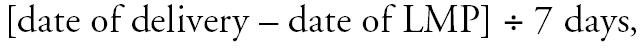


and sonogram-based age as


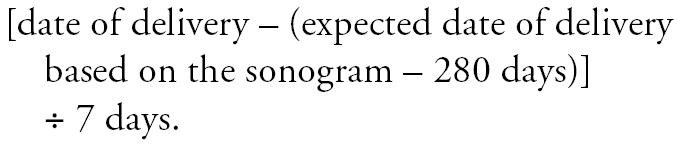


In the Krakow cohort, the reliability of the original gestational age (*n* = 340), relative to LMP-based (*n* = 340) and sonogram-based (*n* = 165) gestational age, was examined through an analysis of internal consistency. In the NYC cohort, the reliability of the original gestational age was compared by creating three mutually exclusive groups – gestational age based on LMP alone (*n* = 90), based on sonogram alone (*n* = 137), or based on both (*n* = 130). Twenty-three NYC subjects, whose method of gestational age calculation is unknown, were not included in this analysis. We assessed the internal consistency of the original variable relative to derived ones by determining Spearman-Brown and Cronbach’s α coefficients, respectively.

Considering the significant ethnic difference in PAH effect observed in our preliminary analysis (Perera et al. 2003) as well as the demographic differences of the mothers (Table 1), we conducted separate analyses for each ethnic group within their full exposure range. Potential confounders identified from the literature were included in the multivariate models if they were related to birth weight, length, or head circumference (*p* < 0.05) or if their absence significantly affected the estimated PAH effect (≥ 10% change) (Greenland 1989).

We also compared the association between the NYC and Krakow cohorts. Because the mean exposure levels between NYC and Krakow cohorts differed by > 10-fold, we selected a subset whose prenatal PAH exposure level lay within the range common to both cohorts (1.80–36.47 ng/m^3^) [*n* =123 African Americans (73%), 161 Dominicans (76%), and 227 Caucasians (67%)]. The possibility that the PAH effects differ according to the gestational age at the time of monitoring was addressed by comparing birth outcome models for the Krakow subjects monitored in the second versus the third trimester. We also examined whether ethnic groups are differentially exposed to other toxicants that are correlated with sociodemographic conditions. Namely, although a common residential pesticide, chlorpyrifos (CPF), has been shown to reduce fetal growth in NYC (Whyatt et al. 2004), pre-natal CPF exposure is believed to be minimal in Krakow (Jedrychowski W, personal communication). We examined the correlation between PAHs and CPF within the NYC cohort, and found it to be low (*p* > 0.1). Furthermore, dropping CPF from the birth weight and birth head circumference models, respectively, did not affect the size of PAH effect for NYC African Americans, indicating that it is unlikely to be a confounder (Perera et al. 2003). Finally, possible residual confounding by each sociodemographic variable shown in Table 1 was considered if it met the criteria for potential cofounders.

In combined analyses, we tested the hypothesis that there is no significant difference in PAH effect across ethnic groups by including indicator variables for the African-American, the Dominican, and interaction terms (NYC African American × PAH, and NYC Dominican × PAH). We considered Caucasians the reference group because our interest lay in quantifying the difference between the Krakow cohort and each NYC ethnic group.

**Figure 1:**
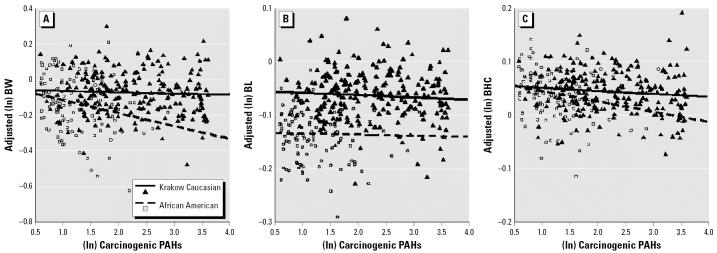
Adjusted scatterplots of birth weight (BW) (*A*), length (BL) (*B*), and head circumference (BHC) (*C*) versus (ln)∑8 c-PAHs, restricted to 1.80–36.47 ng/m^3^ range. The scatterplot was based on the combined multivariate model shown in Table S3(B) (Supplemental Material). The regression lines represent the slopes of the association for the two ethnic groups (123 NYC African Americans and 227 Krakow Caucasians). The scatterplot for NYC Dominicans is not shown because the PAHs had no significant effect on any of the birth outcomes.

The PAH–birth outcome association for each cohort group was visualized by creating adjusted scatterplots for the three birth outcomes. The residual variance on the *y*-axis is plotted against the PAH exposure on the *x*-axis for each ethnic group. The model controlled for (ln) gestational age, newborn sex, maternal prepregnancy weight, height, parity, and delivery fall, winter, and spring, respectively [Table S3(A) in Supplemental Materials (http://www.ehponline.org/docs/2006/8982/suppl.pdf)]. The adjusted scatterplots were generated first for the overall exposure range of each city [Figure S1 in Supplemental Materials (http://www.ehponline.org/docs/2006/8982/suppl.pdf)], and subsequently, within the exposure range common between the two cities (Figure 1). All analyses, including adjusted scatterplots, were conducted in SPSS for Windows (version 11.5; SPSS Inc., Chicago, IL, USA).

## Results

### Descriptive analysis

Table 1 shows the demographic and exposure characteristics of the mothers and the birth outcomes. The 340 Krakow subjects included are similar to those not included (23 women who refused before the onset of the study, 57 women lost to follow-up, and 67 women with incomplete, missing, or poor-quality data) with respect to household income, maternal age, prepregnancy weight, height, ETS exposure, and education (*p* > 0.05) [Table S1(A) in Supplemental Materials (http://www.ehponline.org/docs/2006/8982/suppl.pdf)].

Among 687 fully enrolled NYC women (243 African Americans, 442 Dominicans, 2 with unknown ethnic background), valid PAH data were available for 534 mothers; 10 cases had cord cotinine level > 25 ng/mL; birth weight measurements were available for 448 newborns; birth length measurements were available for 436 newborns; and birth head circumference measurements were available for 425. No significant differences were observed in ∑8 c-PAHs, mother’s age, education level, prepregnancy weight, marital status, daily alcohol intake, frequent intake of PAH-containing foods, and ETS exposure between subjects with missing data and those included [Table S1(B) in Supplemental Materials (http://www.ehponline.org/docs/2006/8982/suppl.pdf)].

In Krakow, prevalence of low birth weight (LBW; < 2,500 g) and preterm birth (< 37 weeks of gestation) were 3.5 and 4.7%, respectively. In NYC, the prevalence of LBW in African-Americans and Dominicans was 6.2 and 2.1%, respectively; and the prevalence of preterm birth was 6.8 and 2.2%, respectively.

Although the study targeted nonsmoking women, we sought to control further for potential confounding by prenatal ETS exposure by using the umbilical cord cotinine level and the women’s self-reported ETS exposure. Between the second- and the third-trimester interviews in the Krakow cohort, the number of smokers at home remained highly consistent (intraclass correlation coefficient = 0.81, *p* < 0.001). The level of prenatal exposure to ETS was low in all three cohorts (Table 1). Among the Krakow cohort, cord cotinine was low, but dose dependent (0.15 ± 0.32 among nonsmokers vs. 0.84 ± 1.60, 0.92 ± 0.93, and 2.62 ± 3.42 ng/mL, among those with one, two, or three smokers at home, respectively) based on the third-trimester questionnaire (Spearman’s rho = 0.391, *p* = 0.006). Cord cotinine level was not dose dependent in African Americans (0.72 ± 1.66 ng/mL with one smoker; 0.31 ± 0.22 ng/mL with two; 0.64 ± 3.56 ng/mL with three; 0.43 ± 0.38 ng/mL with four; Spearman’s rho = 0.16, *p* = 0.25) and in Dominicans (0.33 ± 1.25 ng/mL with one smoker; 0.19 ± 0.35 ng/mL with two; 0.16 ± 0.20 ng/mL with three; Spearman’s rho = 0.072, *p* = 0.63). The mother’s self-reported ETS exposure, the umbilical cord plasma cotinine level, frequent dietary PAH intake, any exposure to alcohol during the entire pregnancy, and daily alcoholic intake were not associated with any of the outcomes in the respective ethnic groups.

In the Krakow cohort, mean LMP-based gestational age was 40 ± 2 weeks (range, 29–43). Mean sonogram-based gestational age was 40 ± 1 (range, 34–45). Cronbach’s α reliability coefficient of the original, LMP-based, and sonogram-based gestational age was 0.90, suggesting a high reliability of the infant’s medical record data. Among the NYC cohort, the reliability of the internal consistency of the original gestational age differed according to the mode of derivation. For those whose gestational age was derived with LMP date (*n* =90), Cronbach’s α and Spearman-Brown was 0.78 and 0.83, respectively. For those whose gestational age was determined by sonogram (*n* = 137), Cronbach’s α and Spearman-Brown reliability coefficients were both 0.90. Among those whose gestational age was estimated using both LMP and sonogram (*n* = 130), both coefficients were 0.94. For the remaining 23 newborns, reliability of the gestational age could not be determined because LMP and sonogram data were missing. Prenatal PAH exposure for the missing group did not differ significantly from the remaining groups (*p*-value for ANOVA > 0.05). As a result, the original gestational age variable was used.

### Risk of prenatal exposure to PAHs on birth weight, birth length, and birth head circumference

To compare the size of the PAH effect between the Krakow and NYC cohorts, we included the same set of confounders—gestational age, baby’s sex, parity, maternal prepregnancy weight, height, and season of delivery—in the final multivariate model (Table 2). Delivery by cesarean section protects against birth head circumference reduction, and was included in the birth head circumference model. Mother’s age, mother’s educational level, any exposures to ETS at home or at work, maternal dietary intake of PAH-containing foods at least twice a week, marital status, any exposure to wine, beer, or hard liquor, respectively, or daily intake of alcohol did not meet the criteria for inclusion in the model. Although particulate matter < 2.5 μm in aerodynamic diameter (PM_2.5_) and season of personal air monitoring might be independent risk factors (Jedrychowski et al. 2004), we could not control for either variable because of the high variance inflation factor induced by these variables.

In the Krakow cohort, prenatal exposure to PAHs was significantly associated with reduced birth weight (β = −0.020, *p* < 0.01; weight decrement = 68.75 g/ln-unit of exposure), birth length (β = −0.009, *p* < 0.01; length decrement = 0.48 cm/ln-unit), and birth head circumference (β = −0.006, *p* = 0.01; head circumference decrement = 0.21 cm/ln-unit) (Table 2). In the NYC cohort prenatal exposure to PAHs was associated with reduced (ln) birth weight (β = −0.06; gram decrement = 177.57 g/ln-unit, *p* < 0.01) among NYC African Americans, but not among NYC Dominican (*p* > 0.1) (Table 2). In the NYC cohort, stratified regression of the PAH effect according to the median CPF value was conducted. For NYC AA, the PAH effect remained similar (β = −0.063 for < median CPF level; β = −0.061 for ≥ median CPF level). As shown in a prior analysis (Perera et al. 2003), exclusion of CPF from the model did not affect the estimated size of PAH effect (change in PAH effect < 3%), indicating that CPF is neither a significant effect modifier nor a confounder.

Prenatal exposure to PAHs was not associated with a significant reduction in gestational age either for the Krakow cohort (β = −0.002, *p* = 0.28) or for the NYC cohort (β for African American = −0.005, *p* = 0.32; β for Dominican = 0.003, *p* = 0.32).

### Comparative analysis of PAH effects between the two cohorts

Table 1 shows that Caucasians and African Americans differ significantly in mother’s age, prepregnancy weight, height, highest completed education, marital status, parity, ETS exposure, dietary PAH intake, any alcohol intake during the current pregnancy, maternal personal PAHs exposure level, and cord cotinine concentration.

In exploratory analyses, both Krakow and NYC samples were restricted to those within the PAH exposure range common between the two cohort studies (1.80–36.47 ng/m^3^). The size of PAH effects for the Krakow cohort were identical (β = −0.021 for birth weight; β = −0.009 for birth length; β = −0.008 for birth head circumference) to the estimates based on the overall range, though not significant (all *p*-values > 0.05) (Table 3). For NYC African Americans within the common range, the effect was larger (β = −0.085, *p* = 0.003 for birth weight; β = −0.023, *p* = 0.03 for birth head circumference) than the estimates based on the cohort’s overall range (0.27–36.47 ng/m^3^) (Table 3). The difference in PAH effect size between the cohorts was determined over the entire range (0.27–272.18 ng/m^3^) [Figure S1 in Supplemental Materials (http://www.ehponline.org/docs/2006/8982/suppl.pdf)] and within the common exposure range (1.80–36.47 ng/m^3^) (Figure 1). The models included relevant covariates, including those that differed between ethnic groups. Across the common exposure range, the effect per ln-unit increase in exposure was significantly greater for African Americans than for Caucasians for birth weight (β for difference in PAH effect = −0.062, *p* = 0.01) and marginally greater for birth head circumference (β for difference in PAH effect = −0.015, *p* = 0.07) (Figure 1). Similarly, across the overall exposure range, the effect of prenatal PAH exposure was significantly greater on the birth weight of African Americans (β for difference in PAH effect for birth weight = −0.043, *p* = 0.01) [Figure S1 and Table S3(A) in Supplemental Materials (http://www.ehponline.org/docs/2006/8982/suppl.pdf)]. The adjusted scatter-plot for birth weight (Figure 1) illustrates that with increasing PAH exposure level, NYC African Americans experienced a significantly greater reduction in birth weight than Caucasians. There were no significant differences in the magnitude of the PAH effect between NYC Dominicans and Caucasians for any of the birth outcomes. For birth length and the birth head circumference, a unit increase in prenatal PAH exposure level did not result in a significantly greater effect in the NYC African Americans.

The effects of prenatal exposure to PAHs on birth weight (β = −0.017, *p* = 0.09), birth length (β = −0.011, *p* < 0.01), and birth head circumference (β = −0.008, *p* < 0.01) among the subset of 202 Krakow mothers who were monitored in the third trimester were very similar to those estimated in the second trimester [Table S4(A) in Supplemental Materials (http://www.ehponline.org/docs/2006/8982/suppl.pdf)]. Furthermore, comparison of maternal demographic characteristics among these participants within the exposure range of interest (1.80–36.47 ng/m^3^) versus those outside (> 36.47 ng/m^3^ for Krakow and < 1.80 ng/m^3^ for NYC samples) demonstrated no significant difference [Table S1(C) in Supplemental Materials (http://www.ehponline.org/docs/2006/8982/suppl.pdf)].

## Discussion

In two separate but parallel prospective cohort studies, *in utero* exposure to airborne PAHs was significantly associated with adverse effects on fetal growth in both Polish Caucasians and U.S. African Americans. There was a signifi-cant association between airborne PAHs and birth outcomes in two locations across a 10-fold exposure range. These findings are of concern because reduced birth weight has been associated with greater risks of fetal and neonatal mortality (Wen et al. 2005), delay in cognitive development (Rice and Barone 2000; Richards et al. 2002), and increased risk of type II diabetes, hypertension, and coronary heart disease during adulthood (Barker 2002).

The two studies targeted women with adequate prenatal care, free from diabetes, hypertension, or HIV, nonsmokers of cigarettes and nonusers of illicit drugs to preclude confounding of the PAH–birth outcome associations. Both Krakow and NYC cohort mothers are representative of the target population. Within both cohorts, the demographic traits of the women who agreed to participate, refused, were lost to follow-up, and remained in the current study are very similar, suggesting that selection bias is unlikely to have occurred. In addition, maternal demographic characteristics of those within the lower exposure range (1.80–36.47 ng/m^3^) were similar to those excluded, demonstrating that selection bias does not account for the apparent difference in PAH–birth outcome between the ethnic groups.

The prevalence of LBW among the current NYC African-American sample was about half of the prevalence generally among U.S. African Americans (7 vs. 13%) (David and Collins 1997). The low incidence of LBW and preterm birth in both cohorts reflect the eligibility criteria and indicate that the women included in the current study are representative of lower-risk pregnant women rather than the general population. As a mounting body of evidence suggests, among pregnant women who have additional risk factors, the risk of prenatal PAH exposure on fetal growth might be considerably greater (Erickson et al. 1999; Wormley et al. 2004).

The observation that there was a significantly greater effect among NYC African Americans than among Krakow Caucasians when the analysis was restricted to a lower common range of exposure suggests increased susceptibility of the former population. The same effect was not seen in NYC Dominicans. We examined whether ETS (both self-reported and cord plasma cotinine), spousal support, educational level, frequent consumption of dietary PAHs, and foreign birth might explain the difference in associations across the ethnic groups. Each of these factors was neither an effect modifier nor a source of confounding in the current analysis. Although we did not ask about dairy, fruit, fish, and vegetable consumption during pregnancy, prenatal vitamin intake was high in all three groups (87% of Dominicans, 96% of African Americans, and 100% of Caucasians). However, neighborhood factors such as social isolation, crime, and poverty were not examined, and these might have confounded the current association. It is also possible that other components of PM_2.5_ or the differential proportion of the carcinogenic PAHs between two cities might have confounded the outcomes.

Other studies have previously observed a greater effect of air pollution experienced by racial minorities and those of lower socioeconomic status (Ito and Thurston 1996; Jerrett et al. 2004). In this analysis, NYC African Americans were of lower socioeconomic status (in education) than Krakow Caucasians and were significantly less likely to be married (hence had less social support). They also had prenatal co-exposure to the fetotoxic pesticide chlorpyrifos (Whyatt et al. 2004), whereas Krakow Caucasians did not. Although lack of adequate prenatal care could not have confounded the outcome, we did not examine history of previous LBW.

Adverse effects seen in NYC African Americans were not evident in NYC Dominicans with comparable prenatal exposure to PAHs. Although the two NYC groups are similar with respect to income and education, they differ significantly in other demographic factors such as current marital status, with NYC Dominican more likely to be married. NYC African Americans also have greater co-exposure to chlorpyrifos (Whyatt et al. 2004). Moreover, NYC African Americans differ from NYC Dominicans in that 77% of the Dominican women were born outside of the United States. Other research suggests that less acculturated Hispanic immigrants might be at lower risk of adverse birth outcomes as well as adulthood morbidities (Flores and Brotanek 2005). Similarly, among low-risk women, prevalence of having an LBW newborn among African-born black women (3.6%) was much lower than for U.S.-born black women (7.5%) (David and Collins 1997). In our cohort study, compared with the newborns from U.S.-born Dominican mothers, the newborns from the recent immigrant Dominican mothers had a higher birth weight (3,466 vs. 3,358 g, *p* = 0.1) and birth length (51.11 vs. 49.62 cm, *p* = 0.003).

The observed differences between ethnic groups may also result from unmeasured contextual factors or from gene–environment interactions (Wiencke 2004). In the present analysis, data on genetic polymorphisms were not available, nor would the sample size permit a meaningful analysis of interactions. In the current analysis, the low number of LBW cases prevented examination of the risk associated with prenatal PAH exposure. Larger studies are needed to determine the genetic, environmental, and/or socioeconomic factors underlying the observed ethnic differences in the effects of transplacental PAH exposure.

Prior studies have suggested that diesel fuel combustion contributes to ambient PAHs in NYC (Tonne et al. 2004), whereas coal burning in small furnaces is a major source of PAHs in Krakow (Lvovsky et al. 2000). We examined whether differences in the proportion of individual PAHs in the air might account for the difference in the birth outcomes by conducting the same regression for each of the eight individual carcinogenic PAHs within the common exposure range. Prenatal exposure to benzo[*g,h,i*]perylene was associated with the largest difference (7-fold) in PAH effect among NYC African Americans compared with Krakow Caucasians. Next were benzo[*b*]fluoranthene and indeno[*1,2,3-c,d*]pyrene, which were each associated with a 5-fold greater effect among African Americans. In NYC, these three PAHs are the most common PAHs (constituting 19, 9, and 10%, respectively, of the eight carcinogenic PAHs). In contrast, in Krakow the relative proportions of each carcinogenic PAH were equal among the eight carcinogenic PAHs. BaP was associated with a 3-fold greater effect for NYC African Americans compared with Krakow Caucasians. BaP was the fourth most common PAH (7%) in NYC, whereas in Krakow, it was the second most common PAH (16%). Prior studies have shown that benzo[*g,h,i*]perylene and ideno[*1,2,3-c,d*]pyrene are predominant PAHs from gasoline combustion (Binková et al. 2003). Embryotoxic activity of dichloromethane extractable organic matter was highest for those collected from the Prague city center with high traffic volume (Binková et al. 2003).

Because PAH effects on fetal growth could differ depending on the timing of exposure (Dejmek et al. 2000), we used Krakow data to consider whether nondifferential exposure misclassification of the exposure during unmeasured gestational months threatens the validity of the PAH–fetal growth association. We observed that indicator variables for season of monitoring are the most significant predictors of the personal exposure level of ∑8 c-PAHs (*R*^2^ = 0.723) in the Krakow cohort (*n* = 340). However, considering the high correlation between seasons of monitoring and the main exposure variable, season of delivery was chosen as an alternative covariate. Multicollinearity of season of delivery and the main exposure was quite low (variance inflation factor of the involved variables < 3). The estimated PAH risk remained identical whether exposure during unmeasured gestational trimester was accounted for by a modeled exposure variable or by indicator variables for delivery season.

Strengths of the study include the validation of self-reported nonsmoking status with cord cotinine level. Positive agreement between ETS and umbilical cord cotinine level in both cohorts precludes PAH exposure misclassification and potential confounding by ETS. A high proportion of Krakow Caucasian women reported that they had at least one glass of wine during the current pregnancy (52%), although the prevalence of daily alcoholic intake was extremely low in all three ethnic groups. Neither “any” exposure to alcohol during the entire pregnancy, “daily” alcoholic intake, nor dietary intake of PAHs had a significant effect on the three birth outcomes.

Mechanisms of fetal toxicity of PAHs are not fully understood but may involve the induction of apoptosis after DNA damage from PAHs, antiestrogenic effects of PAHs, and binding to the human aryl hydrocarbon receptor to induce P450 enzymes or to receptors for placental growth factors, resulting in decreased exchange of oxygen and nutrients (Dejmek et al. 2000).

Given that PAH concentrations in Krakow and NYC are representative of the range seen in much of the developing and industrialized world, reduction of transplacental exposure to PAHs would be expected to have both short-term and long-term benefits to health worldwide. Fossil fuel and wood combustion are the primary sources of ambient PAHs (Bostrom et al. 2002) and are a major source of greenhouse gases that impact the global climate (National Academy of Sciences 2001). It has been estimated that mitigation of these emissions by using readily available technologies would yield substantial health benefits to 3 billion urban residents worldwide (Cifuentes et al. 2001; Levy et al. 2002). The associated economic benefits of reducing fossil fuel–related air pollution are estimated to amount to a savings of many billions of dollars each year in the United States alone (Wong et al. 2004). Thus, policies to reduce ambient concentrations of PAHs should yield substantial benefits to human health and the global environment.

## Figures and Tables

**Table 1:** Demographic and exposure characteristics and pregnancy outcomes of the study cohorts.

Demographic variables	Krakow Caucasians (*n* = 340)	NYC African Americans (*n* = 168)	NYC Dominicans (*n* = 212)
Maternal age (years)	28 ± 4 (18–36)*	24 ± 5 (18–36)	25 ± 5 (18–38)
Prepregnancy weight (kg)	58 ± 9 (40–118)	74 ± 20 (44–149)*	63 ± 13 (40–112)
Maternal height (cm)	165 ± 6 (144–180)	164 ± 9 (132–183)	161 ± 8 (127–178)*
Maternal education [*n* (%)]			
< High school	36 (11)	54 (32)	71 (35)
High school graduate	90 (27)	74 (44)	87 (42)
>High school	214 (63)*	40 (24)	48 (23)
Currently married [*n* (%)]	316 (93)*	21 (13)	66 (31)
Parity [n (%)]	118 (35)	139 (82)*	142 (67)
ETS exposure at home or work [*n* (%)]	131 (39)	88 (52)*	81 (38)
Any alcohol intake [*n* (%)]			
>Wine	175 (52)	13 (8)	21 (10)
>Beer	107 (32)	8 (2)	43 (20)
>Hard liquor	21 (6)	10 (6)	16 (8)
Daily alcohol intake [*n* (%)]	4 (1)	0 (0)	4 (2)
Frequent intake of PAH- containing foods [*n* (%)]	49 (14)	54 (32)*	30 (14)
Taken prenatal vitamin during current pregnancy [*n* (%)]	340 (100)	161 (96)	185 (87)
Household income [*n* (%)]			
< $10,000		64 (38)	99 (47)
$10,000–20,000		48 (29)	53 (25)
$20,001–50,000		47 (28)	29 (14)
> $50,000		3 (2)	17 (8)
Not reported		6 (4)	14 (7)
Polish tax group^a^ [*n* (%)]			
< 37,024 PLZ	229 (67)		
37,024–74,048 PLZ	16 (5)		
> 74,048 PLZ	4 (1)		
Not reported	91 (27)		
Have U.S. Medicaid [*n* (%)]		143 (86)	195 (92)
Maternal personal exposure to ∑8 c-PAHs (ng/m^3^)	39.05 ± 47.63 (1.80–272.18)*	3.34 ± 2.92 (0.52–22.10)	3.72 ± 3.90 (0.27–36.47)
Umbilical cord cotinine (ng/mL)^b^	0.32 ± 0.88 (0.01–8.45) [*n* = 227]	0.36 ± 0.95 (0.01–8.79) [*n* = 122]	0.18 ± 0.87 (0.01–7.75) [*n* = 157]
Newborn sex (female) [*n* (%)]	174 (51)	83 (49)	112 (53)
Birth weight (g)	3,427 ± 493 (1,160–4,700)	3,302 ± 519* (1,875–5,110)	3,439 ± 451 (2,125–4,560)
Birth length (cm)	54.5 ± 3.0 (37.0–64.0)*	50.8 ± 3.1 (34.0–67.5)	50.76 ± 3.1 (19.0–56.5)
Birth head circumference (cm)	33.9 ± 1.5 (26.0–39.0)	33.8 ± 1.8 (28.0–37.5)	34.3 ± 1.3 (29.0–37.5)*
Gestational age (week)^c^	39 ± 2 (29–43)	39 ± 2 (30–42)	40 ± 1 (34–44)*
Cesarean-section delivery [*n* (%)]	64 (19)*	9 (5)	27 (13)

Values are mean ± SD except where indicated.

a For 2005, the mean exchange rate was 1 Euro = 4.2009 Poland Zlotych (PLZ);

b Subjects with completed laboratory analysis as of September 2004.

c Gestational age was calculated using LMP and sonogram data, whenever available.

* Significantly differs from the remaining two groups based on ANOVA followed by Bonferroni correction or pair-wise χ^2^ test.

**Table 2:** Risk of reduced birth outcomes due to prenatal airborne PAH exposure.

	(ln) Birth weight			(ln) Birth length			(ln) Birth head circum		
	β	SE	*p*-Value	β	SE	*p*-Value	β	SE	*p*-Value
Krakow Caucasian (*n* = 340)	Adjusted *R*^2^ = 0.486			Adjusted *R*^2^ = 0.369			Adjusted *R*^2^ = 0.288		
(Constant)	−1.131	0.563	0.046	1.148	0.224	0.000	1.707	0.193	0.000
(ln) ∑8 c-PAHs	−0.020	0.007	0.007	−0.009	0.003	0.003	−0.006	0.002	0.010
Maternal height	0.003	0.001	0.019	0.001	0.000	0.014	0.001	0.000	0.017
Prepregnancy weight	0.003	0.001	0.000	0.001	0.000	0.003	0.001	0.000	0.001
Female sex	−0.059	0.012	0.000	−0.021	0.005	0.000	−0.022	0.004	0.000
(ln) Gestational age	2.365	0.146	0.000	0.721	0.058	0.000	0.445	0.050	0.000
Parity	0.035	0.013	0.008	0.005	0.005	0.307	0.015	0.004	0.001
Fall delivery	−0.040	0.020	0.046	−0.019	0.008	0.018	−0.008	0.007	0.222
Winter delivery	0.007	0.017	0.696	−0.004	0.007	0.597	0.001	0.006	0.909
Spring delivery	0.026	0.019	0.184	0.003	0.008	0.662	0.009	0.006	0.173
Delivery by cesarean section^a^		NA			NA		0.012	0.005	0.031
NYC African American (*n* = 168)	Adjusted *R*^2^ = 0.185			Adjusted *R*^2^ = 0.197			Adjusted *R*^2^ = 0.232		
(Constant)	2.665	1.194	0.027	1.584	0.369	0.000	1.352	0.369	0.000
(ln) ∑8 c-PAHs	−0.055	0.019	0.004	−0.011	0.007	0.112	−0.010	0.007	0.125
Maternal height	0.000	0.001	0.961	0.000	0.000	0.795	0.000	0.000	0.571
Prepregnancy weight	0.001	0.001	0.105	0.000	0.000	0.634	0.000	0.000	0.060
Female sex	−0.042	0.024	0.086	−0.011	0.009	0.196	−0.028	0.009	0.002
(ln) Gestational age	1.474	0.322	0.000	0.632	0.100	0.000	0.578	0.100	0.000
Parity	0.025	0.031	0.408	0.016	0.011	0.159	0.005	0.011	0.634
Fall delivery	−0.008	0.032	0.792	0.002	0.011	0.860	−0.014	0.011	0.221
Winter delivery	−0.003	0.035	0.939	0.002	0.013	0.849	−0.004	0.013	0.737
Spring delivery	−0.034	0.033	0.313	−0.005	0.012	0.665	−0.008	0.012	0.536
Delivery by cesarean section^a^		NA			NA		0.029	0.019	0.118
NYC Dominican (*n* = 212)	Adjusted *R*^2^ = 0.282			Adjusted *R*^2^ = 0.061			Adjusted *R*^2^ = 0.255		
(Constant)	0.610	0.913	0.505	1.548	0.652	0.018	1.599	0.282	0.000
(ln) ∑8 c-PAHs	0.018	0.011	0.094	0.003	0.008	0.712	0.004	0.003	0.168
Maternal height	0.000	0.001	0.757	0.000	0.001	0.573	0.000	0.000	0.490
Prepregnancy weight	0.001	0.001	0.022	0.000	0.000	0.985	−0.000	0.000	0.990
Female sex	−0.023	0.016	0.147	−0.002	0.011	0.853	−0.018	0.005	0.000
(ln) Gestational age	2.013	0.245	0.000	0.629	0.176	0.000	0.518	0.076	0.000
Parity	0.004	0.017	0.795	−0.005	0.012	0.659	−0.005	0.005	0.330
Fall delivery	−0.044	0.023	0.054	0.000	0.016	0.980	−0.005	0.007	0.495
Winter delivery	−0.019	0.024	0.435	0.014	0.017	0.424	0.014	0.007	0.062
Spring delivery	−0.051	0.023	0.029	−0.026	0.017	0.123	−0.005	0.007	0.511
Delivery by cesarean section^a^		NA			NA		0.023	0.008	0.003

Abbreviations: circum, circumference; NA, not applicable.

a Cesarean section delivery method is not a risk factor for birth weight or birth length.

**Table 3:** Risk of reduced birth outcomes, restricting the samples to those within 1.80 – 36.47 ng/m^3^ of PAH exposure range.

	(ln) Birth weight			(ln) Birth length			(ln) Birth head circum		
	β	SE	*p*-Value	β	SE	*p*-Value	β	SE	*p*-Value
Krakow Caucasian									
(Constant)	−1.174	0.758	0.123	0.929	0.303	0.002	1.636	0.259	0.000
(ln) ∑8 c-PAHs	−0.021	0.013	0.091	−0.009	0.005	0.067	−0.008	0.004	0.059
Maternal height	0.004	0.002	0.015	0.002	0.001	0.002	0.001	0.001	0.023
Prepregnancy weight	0.003	0.001	0.003	0.000	0.000	0.222	0.001	0.000	0.094
Female sex	−0.056	0.016	0.001	−0.020	0.006	0.002	−0.018	0.005	0.001
(ln) Gestational age	2.338	0.197	0.000	0.750	0.079	0.000	0.459	0.067	0.000
Parity	0.045	0.016	0.006	0.011	0.007	0.103	0.020	0.005	0.000
Fall delivery	−0.040	0.022	0.070	−0.018	0.009	0.038	−0.011	0.007	0.126
Winter delivery	0.020	0.021	0.334	0.004	0.008	0.633	−0.001	0.007	0.941
Spring delivery	−0.022	0.033	0.502	−0.013	0.013	0.331	−0.005	0.011	0.682
Delivery by cesarean section		NA			NA		0.015	0.007	0.031
NYC African American									
(Constant)	2.348	1.360	0.087	1.785	0.426	0.000	1.603	0.515	0.002
(ln) ∑8 c-PAHs	−0.085	0.028	0.003	−0.008	0.009	0.341	−0.023	0.011	0.029
Maternal height	−0.002	0.002	0.289	0.000	0.000	0.323	0.000	0.001	0.664
Prepregnancy weight	0.001	0.001	0.276	0.000	0.000	0.378	0.000	0.000	0.215
Female sex	−0.064	0.030	0.034	−0.010	0.009	0.285	−0.042	0.012	0.000
(ln) Gestational age	1.650	0.370	0.000	0.598	0.116	0.000	0.541	0.140	0.000
Parity	0.022	0.039	0.574	0.013	0.012	0.278	0.005	0.016	0.739
Fall delivery	0.042	0.041	0.315	0.020	0.013	0.125	−0.008	0.016	0.631
Winter delivery	0.007	0.040	0.861	0.009	0.013	0.496	−0.001	0.016	0.929
Spring delivery	−0.004	0.039	0.923	0.011	0.012	0.359	−0.005	0.015	0.722
Delivery by cesarean section		NA			NA		0.043	0.023	0.061
NYC Dominican									
(Constant)	1.772	1.150	0.125	1.376	0.879	0.119	1.926	0.332	0.000
(ln) ∑8 c-PAHs	0.006	0.017	0.709	0.011	0.013	0.404	0.005	0.005	0.289
Maternal height	0.000	0.001	0.874	0.001	0.001	0.448	0.000	0.000	0.941
Prepregnancy weight	0.002	0.001	0.024	0.000	0.001	0.977	0.000	0.000	0.287
Female sex	−0.014	0.019	0.456	0.007	0.014	0.631	−0.016	0.005	0.002
(ln) Gestational age	1.701	0.308	0.000	0.658	0.236	0.006	0.434	0.089	0.000
Parity	0.004	0.021	0.833	−0.006	0.015	0.675	−0.006	0.006	0.329
Fall delivery	−0.054	0.029	0.064	−0.005	0.022	0.805	−0.004	0.008	0.614
Winter delivery	−0.010	0.029	0.719	0.018	0.022	0.410	0.012	0.008	0.142
Spring delivery	−0.062	0.028	0.027	−0.025	0.021	0.229	−0.009	0.008	0.244
Delivery by cesarean section		NA			NA		0.019	0.008	0.024

Abbreviations: circum, circumference; NA, not applicable.
