# Supplementary material for: International Studies of Prenatal Exposure to Polycyclic Aromatic Hydrocarbons and Fetal Growth
Source: Environ Health Perspect. 2006 Aug 3;114(11):1744–50. doi: 10.1289/ehp.8982 (PMC1665416; doi:10.1289/ehp.8982)
Supplement: Supplemental Figures and Tables [file ehp0114-001744s1.pdf]

Supplemental Materials

Table S1(A). Demographic/exposure characteristics of Krakow women at study initiation and currently

	Eligible women At study initiation		Current Sample		
	Agreed (N = 505)	Refused (N = 23)	Remained (N=448)	Lost to follow-up (N=57)	Current sample (N=340)
	Mean ± SD [Range]				
Maternal age (years)	28 ± 4 [18 – 36]	28 ± 4 [21 – 35]	28 ± 4 [18 – 36]	28 ± 4 [18 – 35]	28 ± 4 [18 - 36.07]
Maternal height (cm)	165 ± 5 [144 – 180]	167± 6 [156 – 185]	165 ± 5 [144 –78]	166 ± 6 [152-180]	165 ± 6 [144 - 180]
Pre-pregnancy weight (kg)	58 ± 8 [40 – 118]	58 ± 9 [43 – 78]	58 ± 9 [40 – 118]	57 ± 6 [45 – 76]	58 ± 9 [40 - 118]
Parity (Yes)	317 (62.8%)	14 (60.9%)	278 (62.1%)	39 (68.4%)	118 (34.7%)*
Maternal Education					
< High School[1]	49 (9.7%)	1 (4.3%)	42 (9.4%)	7 (12.3%)	36 (10.6%)
HS/Tech School	126 (25%)	7 (30.4%)	109 (24.3%)	17 (29.8%)	90 (26.5%)
> High School	330 (65.3%)	15 (65.2%)	297 (66.3%)	33(57.9%)	214 (62.9%)
Income tax group					
<37,024 Poland Zlotych(PLZ)			305 (68.1%)	38(66.7%)	229(67.4%)
37,024 – 74,048 PLZ			20 (4.5%)	2(3.5%)	16(4.7%)
> 74,048 PLZ			5 (1.1%)	0(0%)	4(1.2%)
Not reported			118 (26.3%)	17 (29.8%)	91(26.8%)
Regularly smoked before pregnancy[2]	137(27.1%)	13 (56.5%)**	118 (26.3%)	19 (33.3%)	101 (29.6%)
ETS at home/work[3]	174 (34.5%)	9 (39.1%)	150 (33.5%)	24 (42.1%)	131 (38.5%)
Occasional alcohol intake[4]	298 (59%)	19 (82.6%)*	264 (58.9%)	34 (59.6%)	n.a.
Daily Alcohol intake	n.a.	n.a.	n.a.	n.a.	4 (1.2%)
Ate smoked foods 1-2 times/week[9]	153 (30.3%)	6 (26.1%)	129 (28.8%)	24 (42.1%)	102 (29.9%)

NOTE:
[1] Includes primary school and vocational school (11 years of education). [2]History of ever smoking at least 1 cigarette a day for at least a 6 month period during lifetime prior to the current pregnancy. [3]Percent with at least one smoker at home or at work. [4]Percent who reported drink "occasionally" during the current pregnancy. **$p < 0.01$ between the given vs. other groups. * $p < 0.05$ between the given vs. other groups.

Table S1(B). Demographic characteristics of women who are not included in current analysis versus included women in New York City cohort study.

	Included subjects		Not included subjects	
	N	Mean ± S.D.	N	Mean ± S.D.
∑8 c-PAHs (ng/m^3)	380	3.55 ± 3.50	166	3.39 ± 5.17
Mother's age (years)	379	25 ± 5	304	25 ± 5
Pre-pregnancy weight (kg)	380	68 ± 17	292	66 ± 17
Maternal education [n (%)]	380		307	
< HS		125 (32.9%)		109 (35.5%)
HS graduate		161 (42.4%)		119 (38.8%)
Attained > HS		88 (23.2%)		71 (23.1%)
Refused / missing		6 (1.6%)		8 (2.6%)
Currently married [n (%)]	380	87 (23.7%)	307	94 (30.6%)
Frequent intake of PAH containing foods [n (%)]	380	83 (21.8%)	307	53 (17.3%)
Daily alcohol intake [n (%)]	380	4 (1.1%)	307	3 (1.0%)
ETS at home/work[n (%)]	380	168 (44.2%)	307	103 (33.6%)

Table S1 (C). Maternal Demographic Characteristics of Those Included in Comparative Analysis (PAH exposure range within 1.80 – 36.47 ng/m^3) versus Those Excluded (> 36.47 ng/m^3 for Krakow and <1.80 ng/m^3 for NYC samples)

	NYC Dominican		NYC African-American		Krakow Caucasian	
	Not Included	Included	Not Included	Included	Not Included	Included
Education						
< HS	20(39.2%)	51(32.9%)	14(30.4%)	40(32.8%)	17(14.8%)	19(8.4%)
HS graduate	17(33.3%)	70(45.2%)	18(39.1%)	56(45.9%)	36(31.3%)	54(24.0%)
Attained > HS	14(27.5%)	34(21.9%)	14(30.4%)	26(21.3%)	62(53.9%)	152(67.6%)
ETS, home or work	20(39.2%)	61(37.9%)	20(43.5%)	67(54.9%)	46(40.0%)	85(37.8%)
Currently married	14(27.5%)	52(32.5%)	7(15.2%)	14(11.5%)	99(86.1%)	217(96.4%)
Household income						
< $10,000	25(54.3%)	74(48.7%)	13(29.5%)	51(43.2%)		
$10,001 - 20,000	10(21.7%)	43(28.3%)	15(34.1%)	33(28.0%)		
$20,001 - 50,000	6(13.0%)	23(10.3%)	14(31.8%)	33(28.0%)		
$ >50,000	5(10.9%)	12 (7.9%)	2 (4.5%)	1 (0.8%)		
Not reported	0 (0%)	0 (0%)	0 (0%)	0 (0%)		
Polish income-tax						
< 37,024 PLZ					76(66.1%)	153(68.0%)
37,024 – 74,048 PLZ					6(5.2%)	10(4.4%)
> 74,048 PLZ					2(1.7%)	2(0.9%)
Not reported					31(17.0%)	60(26.7%)

Table S2. Correlation coefficient between actual versus predicted personal PAH exposure.

	1st Personal monitoring	2nd Personal monitoring	3rd Personal monitoring	Predicted overall mean	Predicted mean, 1st trimester	Predicted mean, 2nd trimester	Predicted mean, 3rd trimester
1st Personal monitoring (N)	1.000 (72)	0.157 (72)	-0.459** (67)	0.807** (72)	0.570** (72)	0.733** (72)	-0.393** (72)
2nd Personal monitoring [a] (N)		1.000 (340)	0.512** (68)	0.472** (340)	-0.488** (340)	0.608** (340)	0.634** (340)
3rd Personal monitoring (N)			1.000 (68)	-0.331** (68)	-0.796** (68)	-0.106 (68)	0.688** (68)
Predicted overall mean (N)				1.000 (340)	0.332** (340)	0.896** (340)	0.040 (340)
Predicted mean, 1st trimester (N)					1.000 (340)	0.095 (340)	-0.841** (340)
Predicted mean, 2nd trimester (N)						1.000 (340)	0.119* (340)
Predicted mean, 3rd trimester (N)							1.000 (340)

NOTE:
** Correlation is significant at the 0.01 level (2-tailed).
* Correlation is significant at the 0.05 level (2-tailed).

Table S3 (A). Multiple linear regression of the combined data over the entire exposure range.

	(ln)BW			(ln)BL			(ln)BHC		
	β	se	p-value	β	se	p-value	β	se	p-value
(Constant)	0.348	0.458	0.448	1.446	0.211	0.000	1.657	0.150	0.000
(ln) Σ8 c-PAHs[a]	-0.007	0.006	0.241	-0.003	0.003	0.361	-0.004	0.002	0.041
Mother's height	0.001	0.001	0.093	0.001	0.000	0.056	0.000	0.000	0.033
Pre-pregnancy weight	0.001	0.000	0.000	0.000	0.000	0.221	0.000	0.000	0.001
Newborn gender	-0.043	0.009	0.000	-0.014	0.004	0.001	-0.022	0.003	0.000
Gestational age	2.057	0.122	0.000	0.668	0.056	0.000	0.485	0.040	0.000
Parity	0.020	0.010	0.054	0.002	0.005	0.636	0.007	0.003	0.043
Delivered in the fall	-0.025	0.013	0.067	-0.005	0.006	0.417	-0.009	0.004	0.055
Delivered in winter	-0.003	0.014	0.848	0.002	0.006	0.738	0.002	0.005	0.688
Delivered in the spring	-0.019	0.014	0.154	-0.012	0.007	0.068	-0.001	0.005	0.832
Indicator for NYCAA	-0.028	0.026	0.297	-0.070	0.013	0.000	-0.004	0.009	0.622
Indicator for NYCD	-0.045	0.024	0.060	-0.085	0.012	0.000	-0.008	0.008	0.350
(ln)Σ8 c-PAHs × NYCAA[b]	-0.043	0.016	0.007	-0.006	0.008	0.416	-0.008	0.005	0.150
(ln)Σ8 c-PAHs × NYCD[b]	0.023	0.013	0.066	0.005	0.006	0.374	0.009	0.004	0.042
C-section delivery		N.A.			N.A.		0.015	0.005	0.001

NOTE:
[a] denotes the effect of (ln)Σ 8 c-PAHs on the reference group (KC). [b] denotes difference in PAH effect of NYCAA or NYCD, respectively, compared to KC.

Table S3 (B). Multiple linear regression of the combined data, restricting to the common PAH exposure range (1.80 – 36.47 ng/m^3).

	Birth weight			Birth length			Birth head circumference		
	β	se	p-value	β	se	p-value	β	se	p-value
(Constant)	0.648	0.577	0.262	1.388	0.285	0.000	1.763	0.196	0.000
(ln) Σ8 c-PAHs	-0.010	0.012	0.410	-0.004	0.006	0.445	-0.007	0.004	0.094
Mother's height	0.001	0.001	0.431	0.001	0.000	0.115	0.000	0.000	0.373
Pre-pregnancy weight	0.001	0.000	0.009	0.000	0.000	0.607	0.000	0.000	0.026
Newborn gender	-0.041	0.012	0.000	-0.010	0.006	0.095	-0.023	0.004	0.000
(ln) Gestational age	2.002	0.153	0.000	0.685	0.076	0.000	0.470	0.052	0.000
Parity	0.024	0.013	0.057	0.003	0.006	0.600	0.009	0.004	0.033
Delivered in the fall	-0.021	0.016	0.200	-0.006	0.008	0.495	-0.009	0.005	0.102
Delivered in winter	0.008	0.016	0.626	0.008	0.008	0.333	0.001	0.005	0.842
Delivered in the spring	-0.032	0.018	0.072	-0.015	0.009	0.090	-0.008	0.006	0.169
Indicator for NYCAA	0.004	0.042	0.915	-0.081	0.020	0.000	0.006	0.014	0.652
Indicator for NYCD	-0.029	0.038	0.450	-0.102	0.019	0.000	-0.011	0.013	0.400
(ln)Σ8 c-PAHs × NYCAA	-0.062	0.025	0.014	0.002	0.012	0.854	-0.015	0.008	0.072
(ln)Σ8 c-PAHs × NYCD	0.014	0.022	0.525	0.016	0.011	0.139	0.011	0.007	0.132
C-section delivery		N.A.			N.A.		0.017	0.006	0.004

Table S4(A). Risk of reduced birth outcomes among KC, who were monitored during the third trimester.

	Birth weight			Birth length			Birth head circumference		
	β	se	p-value	β	se	p-value	β	se	p-value
(Constant)	0.392	0.844	0.643	1.860	0.319	0.000	2.162	0.274	0.000
(ln) Σ8 c-PAHs	-0.017	0.010	0.089	-0.011	0.004	0.003	-0.008	0.003	0.009
Mother's height	0.002	0.002	0.210	0.000	0.001	0.777	0.001	0.000	0.278
Pre-pregnancy weight	0.004	0.001	0.000	0.001	0.000	0.000	0.001	0.000	0.001
Newborn gender	-0.064	0.016	0.000	-0.023	0.006	0.000	-0.024	0.005	0.000
Gestational age	1.971	0.220	0.000	0.567	0.083	0.000	0.338	0.071	0.000
Parity	0.034	0.017	0.041	0.006	0.006	0.366	0.017	0.005	0.002
Delivered in the fall	-0.028	0.026	0.270	-0.013	0.010	0.192	-0.011	0.008	0.181
Delivered in the winter	0.007	0.024	0.783	-0.008	0.009	0.354	0.002	0.008	0.759
Delivered in the spring	0.031	0.026	0.249	0.008	0.010	0.426	0.015	0.008	0.079
C-section delivery		N.A.			N.A.		0.013	0.007	0.071

Table S4 (B). Demographic characteristics of KC subjects who were monitored in the third vs. the second trimester.

	Personally air monitored in the 2nd trimester (N=137)	Personally air monitored in the 3rd trimester (N=202)
	Mean ± S.D.	Mean ± S.D.
Σ 8 c-PAHs (ng/m^3)	37.94 ± 49.45	39.68 ± 46.56
Gestational age at delivery (weeks)	39.39 ± 1.85	39.41 ± 1.39
Birth weight (g)	3440.37 ± 521.31	3422.03 ± 473.52
Birth length (cm)	54.60 ± 3.31	54.49 ± 2.74
Birth head circumference (cm)	33.98 ± 1.59	33.89 ± 1.41
Mother's age (years)	28.18 ± 3.45	28.05 ± 3.92
Mothers height (cm)	165.32 ± 5.86	164.86 ± 5.47
Pre-pregnancy weight (kg)	59.12 ± 8.43	57.94 ± 9.54
Maternal education		
< High school (%)	11(8%)	25(12%)
High School equivalent (%)	37(27%)	52(38%)
>High School (%)	89(65%)	125(62%)
ETS exposure (% yes)	51(37%)	79 (39%)
Marital status (% yes)	130(95%)	185(92%)
Parity (% yes)	47(34%)	70(35%)
Daily alcohol intake (% yes)	1(0.7%)	3(1.5%)
High dietary PAH intake (% yes)	17(12%)	31(15%)
Gender (% female)	67(49%)	106(52%)
C-section delivery (% yes)	33(24%)	31(15%)

Figure S1. Regression model adjusted scatter-plots of birth outcomes vs. (ln) of total PAHs over the entire exposure range in the two cities.

The scatter plot was based on the adjusted birth weight value in (ln) scale based on the regression coefficients shown in Table S3A, and includes (ln) gestational age, newborn gender, maternal pre-pregnancy weight and height, parity, birth season as the fall, winter and spring. The regression lines represent the slopes of the association for the two ethnic groups, for whom PAH effect was significant. The PAH effect on birth weight was significant for KC (p-value <0.01) and NYC AA (p-value <0.01), and significantly greater for NYC AA (p-value = 0.007) for birth weight. The PAH effect on birth length was significant for KC (P-value <0.01), but not for NYC AA (p-value = 0.11). The PAH effect on the birth head circumference was significant for KC (p = 0.01), but not for the NYC AA (p = 0.1). One high value for NYC African-Americans on the birth head circumference was not included in the graph to improve legibility. The exclusion of this value did not materially change the outcome. The scatter plot for NYC Dominicans is not shown because the PAHs had no significant effect on the birth outcomes.

(A).

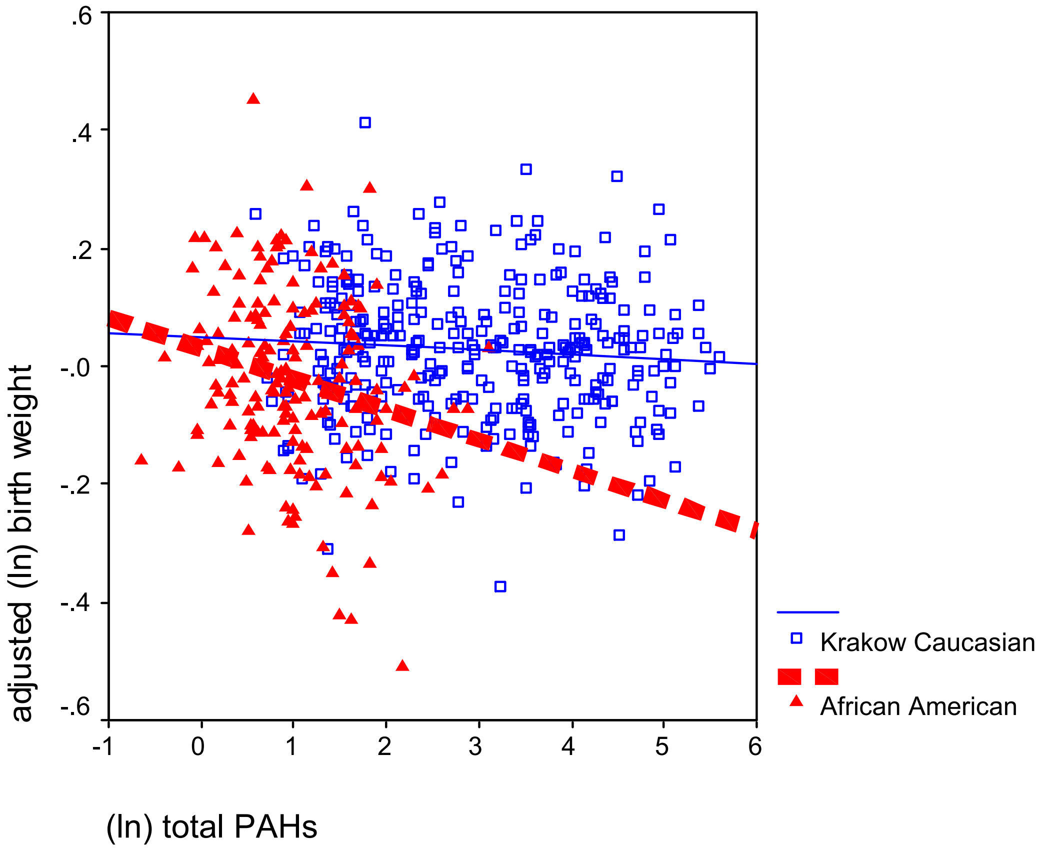
.6
.4
.2
-.0
-.2
-.4
-.6
adjusted (ln) birth weight
-1
0
1
2
3
4
5
6
Krakow Caucasian
African American
(ln) total PAHs

(B).

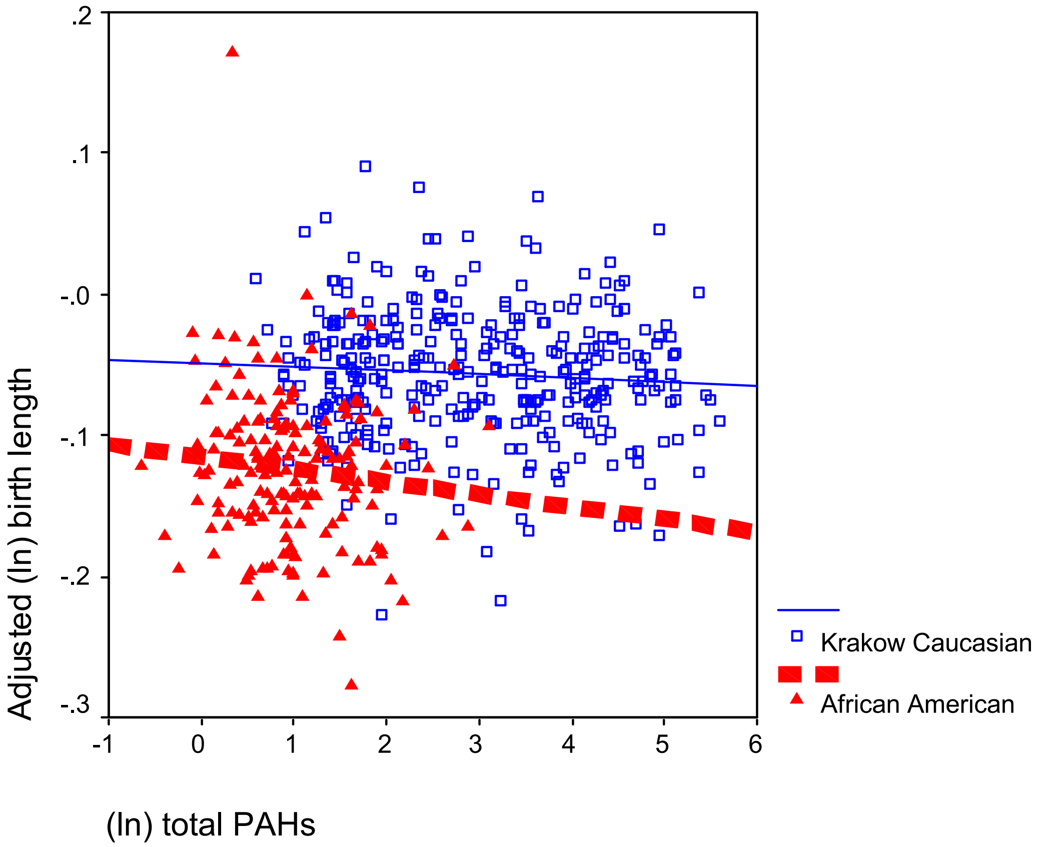

.2
.1
-.0
-.1
-.2
-.3
Adjusted (ln) birth length
-1
0
1
2
3
4
5
6
(ln) total PAHs
Krakow Caucasian
African American

(C).

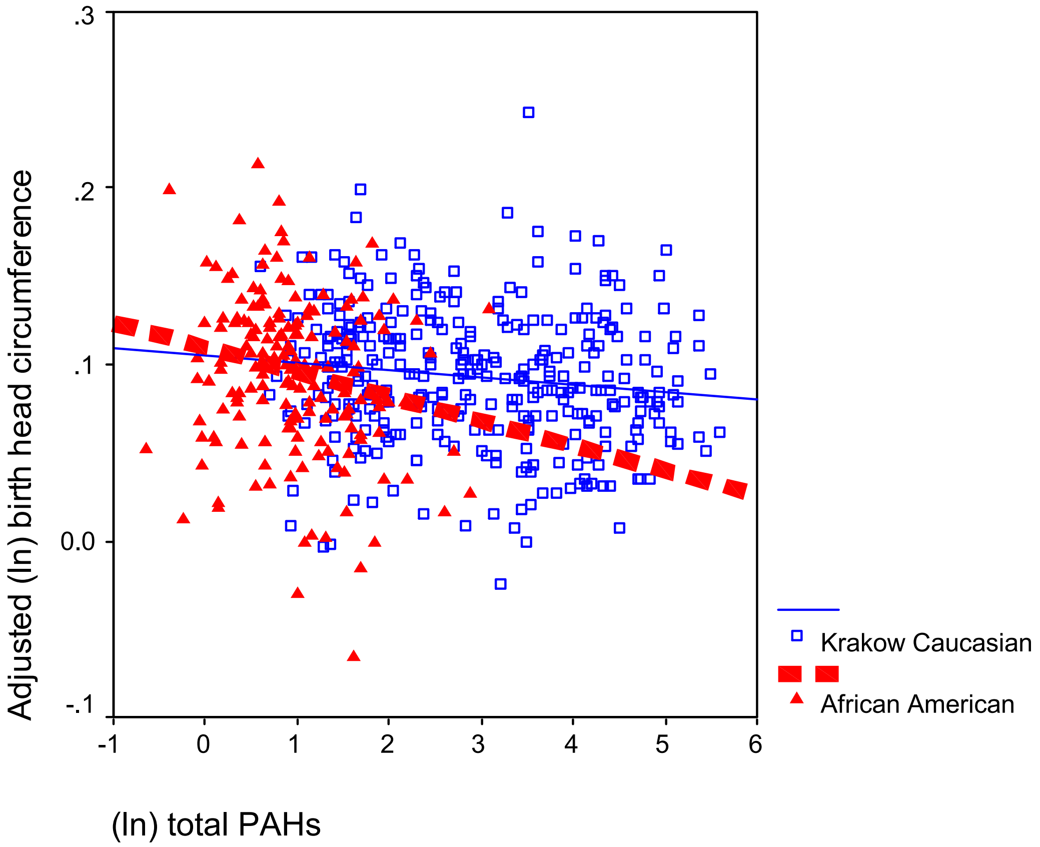

.3
.2
.1
0.0
-.1
Adjusted (ln) birth head circumference
-1
0
1
2
3
4
5
6
Krakow Caucasian
African American
(ln) total PAHs
